# Chloroplast genome organization and phylogeny of *Gynochthodes cochinchinensis* (DC.) Razafim. & B. Bremer (Rubiaceae)

**DOI:** 10.1080/23802359.2020.1862716

**Published:** 2021-01-27

**Authors:** Mary Ann C. Bautista, Wenqin Tao, Yan Zheng, Yunfei Deng, Tao Chen, Shenyu Miao

**Affiliations:** a South China Botanical Garden, Chinese Academy of Sciences, Guangzhou, China; b Shenzhen Fairy Lake Botanical Garden, Chinese Academy of Sciences, Shenzhen, China; c University of Chinese Academy of Sciences, Beijing, China; d School of Life Sciences, Guangzhou University, Guangzhou, China

**Keywords:** *Gynocthodes cochinchinensis*, Rubiaceae, complete chloroplast genome, phylogeny

## Abstract

*Gynochthodes cochinchinensis* previously known as *Morinda cochinchinensis* is considered as potential medicinal plant in family Rubiaceae. In this paper, the complete chloroplast genome of *G. cochinchinensis* was sequenced and characterized for the first time. The cp genome of *G. cochinchinensis* was 153,022 bp in length containing a large single copy region (83,799 bp), a small single copy region (17,591 bp), and a pair of inverted repeat regions (25,816 bp). It has a total of 131 genes, comprising of 86 protein-coding genes, eight rRNA genes, and 37 tRNA genes. Phylogenetic analysis revealed that *Gynochthodes cochinchinensis* together with *Gynochthodes officinalis* were closely related to genus *Morinda*.

The genus *Gynochthodes* Blume under tribe Morindeae (Rubiaceae) is composed mostly of lianoid species characterized with inflorescence that has 8–9 flowers per umbel, globose stipule, umbilicate drupe and 4-locular ovary (Blume 1827). Currently, there are 93 known species and they are widely distributed in tropical Asia, Australasia and Madagascar (Razafimandimbison and Bremer 2011). The increase in number of recognized species can be attributed to the wider circumscription of *Gynochthodes* proposed by Razafimandimbison et al. (2009). The majority of lianescent species of *Morinda* L. with small flowers have been transferred to *Gynochthodes*. In this case, the Chinese species *Morinda cochinchinensis* DC. is now accepted as *Gynochthodes cochinchinensis* (DC.) Razafim. & B. Bremer.

Morphologically, *G. cochinchinensis* is quite distinct by the presence of branches with persistent leafless stipules and densely ferruginous or yellow villosulous exterior when young. It is also occasionally used by the tribes in Similipal Biosphere Reserve for its medicinal properties and belief that it can reduce body weight (Pharmacognocy 2020). However, no recent studies, either taxonomic or pharmacological have been conducted focusing *G. cochinchinensis*. Hence, we sequenced and characterized the complete chloroplast genome of *G. cochinchinensis* to provide basic genetic information that can help in resolving the phylogenetic relationships within Tribe Morindeae.

Total genomic DNA was extracted using modified CTAB method from the fresh leaves collected from Longshitou Village, Longhua Town, Longmen County, Guangdong Province, China (E114°13′02.59″; N23°32′15.14″). The voucher specimen (20181208014) was deposited in the herbarium of Shenzhen Fairy Lake Botanical Garden (SZG). Short-insert sized libraries (350 bp) were constructed using Nextera XT DNA Library Preparation Kit. High-throughput sequencing was performed in Illumina Novseq 600 platform producing paired-end sequences with an average read length of 150 bp. The raw Illumina reads were filtered using NGS-QC toolkit (Patel and Jain 2012) then the high-quality reads were assembled into contigs via the de novo assembler SPAdes 3.11.0 (Vasilinetc et al. 2015). Annotations were conducted using CpGAVAS (Liu et al. 2012), RNAmmer 1.2 Server (Lagesen et al. 2007) and tRNAscan-SE (Chan and Lowe 2019) for the protein-coding genes, rRNA and tRNA.

The complete chloroplast genome of *G. cochinchinensis* (MW026443) has a total length of 153,022 bp with an average sequencing depth of 2959.9X. It has a typical quadripartite structure containing a large single copy region (83,799 bp) and a small single copy region (17,591 bp), separated by two inverted repeat regions (25,816 bp). A total of 131 genes were identified, including 86 protein-coding genes, eight rRNA genes, and 37 tRNA genes. Among the observed genes, 15 genes (*trn*K-UUU, *rps*16, *trn*G-UCC, *atp*F, *rpo*C1, *trn*L-UAA, *trn*V-UAC, *pe*tB, *pet*D, *rpl*16, *rpl*2, *ndh*B, *trn*I-GAU, *trn*A-UGC, *ndh*A) contained single intron while *clp*P and *ycf*3 genes have two introns. The *rps*12 was also trans-spliced, having the 5′ terminal in the LSC region and 3′ end duplicated in the IR regions. Overall GC content was 38.1%, nearly identical to GC content of other species from Morindeae.

Phylogenetic reconstruction was conducted using RAxML 8.2.11 with the GTR + I + G nucleotide substitution model (Stamatakis 2014). The complete cp genome of *G. cochinchinensis* was aligned with the other 23 species of family Rubiaceae. The bootstrap consensus tree inferred from 1000 replicates using *Apocynum venetum* as an outgroup revealed that *G. cochinchinensis* was closest to *Gynochthodes officinalis* (Figure 1). Both species have a sister-group relationship to *Morinda citrifoli*a. This is not surprising since genera in Morindeae such as *Morinda* and *Gynochthodes* are morphologically distinct by the presence of head inflorescences and synacarpous fruits (Razafimandimbison et al. 2009). Together with other woody species of subfamily Rubioideae (Rubiaceae), *G. cochinchinensis* was within the Psychotrieae alliance clade.

**Figure 1.**
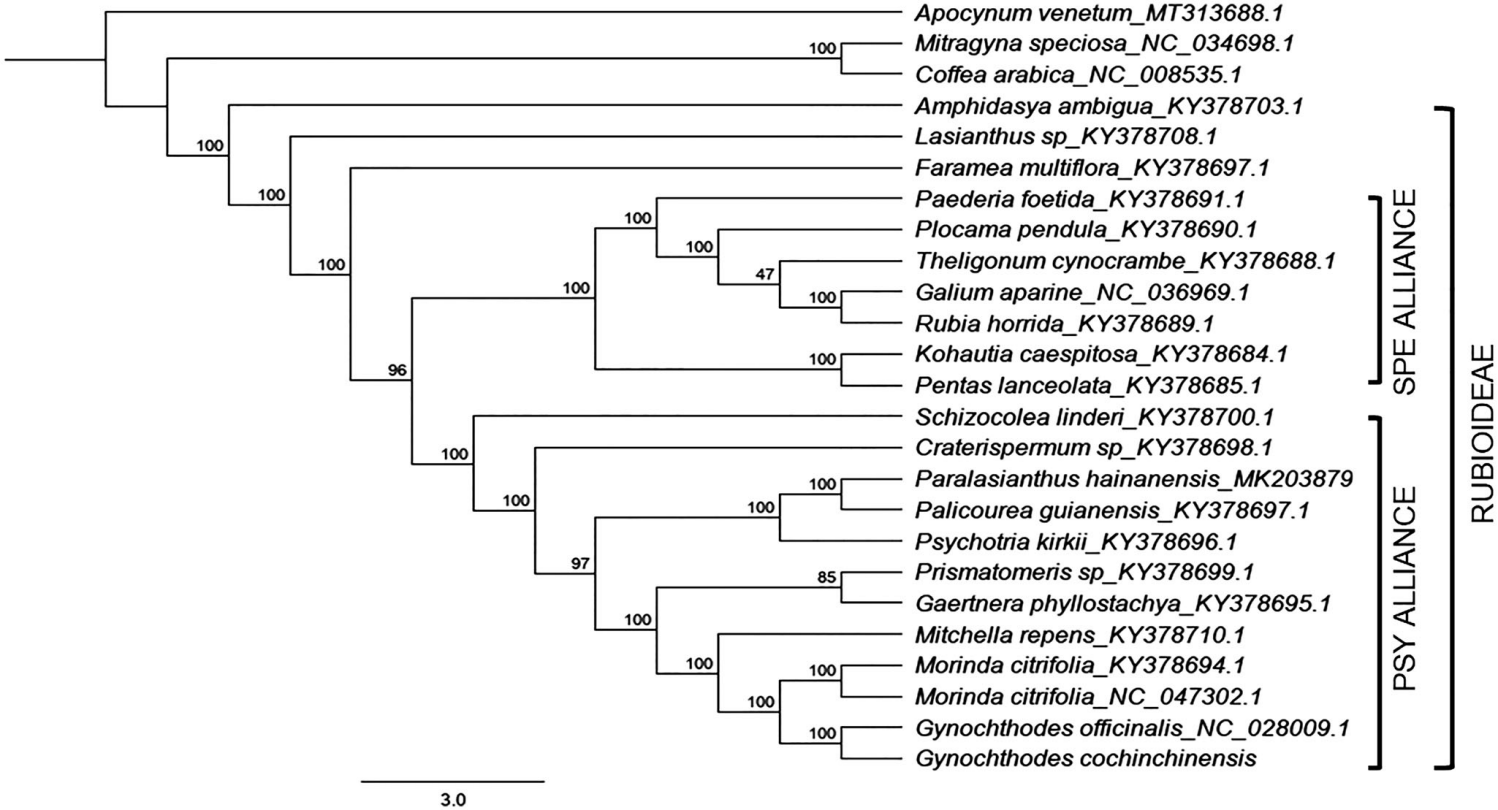
Maximum likelihood (ML) tree reconstruction of 24 taxa in Rubiaceae based on 75 shared CDS in the chloroplast genomes. Numbers on each nodes represent the bootstrap support.

## Data Availability

The complete chloroplast genome sequence of *Gynochthodes cochinchinensis* was deposited in NCBI GenBank with the accession number MW026443. Raw sequencing data were also added in SRA database with the BioSample accession number SAMN16559770 and BioProject accession number PRJNA672143 (https://www.ncbi.nlm.nih.gov/nuccore/MW026443; https://www.ncbi.nlm.nih.gov/sra/PRJNA672143)
